# The roles of ASK family proteins in stress responses and diseases

**DOI:** 10.1186/1478-811X-7-9

**Published:** 2009-04-24

**Authors:** Kazuki Hattori, Isao Naguro, Christopher Runchel, Hidenori Ichijo

**Affiliations:** 1 Laboratory of Cell Signaling, Graduate School of Pharmaceutical Sciences, The University of Tokyo, 7-3-1 Hongo, Bunkyo-ku, Tokyo 113-0033, Japan

## Abstract

Apoptosis signal-regulating kinase 1 (ASK1) is a member of the mitogen-activated protein kinase kinase kinase family, which activates c-Jun N-terminal kinase and p38 in response to a diverse array of stresses such as oxidative stress, endoplasmic reticulum stress and calcium influx. In the past decade, various regulatory mechanisms of ASK1 have been elucidated, including its oxidative stress-dependent activation. Recently, it has emerged that ASK family proteins play key roles in cancer, cardiovascular diseases and neurodegenerative diseases. In this review, we summarize the recent findings on ASK family proteins and their implications in various diseases.

## Introduction

**Figure 1:**
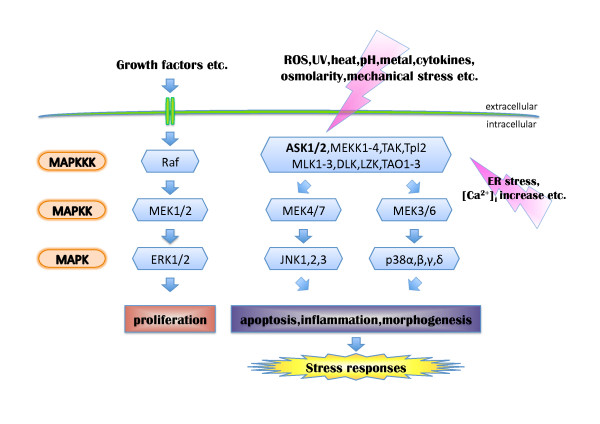
**The mammalian MAP kinase cascade**. ASK1 and ASK2 belong to the MAPKKK family, whose members respond to various external stimuli and initiate the MAPK cascade. There are at least 20 MAPKKKs in vertebrates that selectively phosphorylate and activate MAPKKs leading to a specific activation profile of MAPK family members. The ASK family can activate the JNK and p38 pathways but not the ERK pathway.

All living organisms are exposed to numerous physicochemical stressors during their lifetime, and appropriate responses to stress at the cellular level are essential for the maintenance of homeostasis. The mitogen-activated protein kinase (MAPK) cascades are thought to be crucial among the major signaling pathways that regulate cellular stress responses. However, the mechanisms by which cells sense stresses and convert information about them into cellular signals are poorly understood. The MAPK pathway consists of a cascade of three protein kinases (Figure 1). These protein kinases are sequentially activated, such that the MAPK kinase kinase (MAPKKK) phosphorylates and activates the MAPK kinase (MAPKK), which in turn phosphorylates and activates the MAPK. MAPKs control a wide variety of cellular functions, including proliferation, differentiation, migration and apoptosis. Considering that some MAPKKKs are capable of activating multiple MAPK modules or activating the same modules for different durations, the diversity of the MAPKKKs may have evolved to allow cells to integrate specific MAPK pathways [1]. On the other hand, it has been revealed that a number of diseases, such as cancer, cardiovascular diseases and neurodegenerative diseases, are intimately related to stress response mechanisms mediated by MAPK cascades. In this review, we focus on the regulatory mechanisms of ASK family proteins and their relations to human diseases.

### Regulation of ASK1 activity

**Figure 2:**
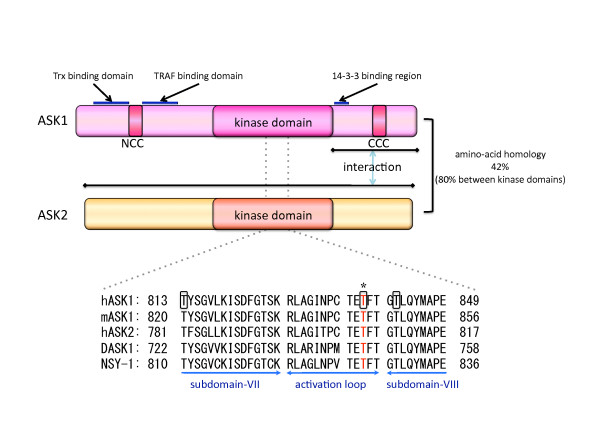
**Schematic illustrations of ASK1 and ASK2**. The domain structures of ASK1 and ASK2 are illustrated. The binding domains of Trx and TRAF exist in the N-terminus of ASK1. Two coiled-coil domains (NCC and CCC) are important for the homomeric interaction and the activation of ASK1. ASK2 associates with the ASK1 C-terminus, which is required to stabilize the ASK2 protein. In the lower part, highly conserved amino acid sequences around the activation loop of the kinase domains of the ASK family are aligned with orthologues of invertebrate ASK1 (DASK1: *D. melanogaster*, NSY-1: *C. elegans*). A phosphorylation site essential for ASK1 activation (Thr845 in mouse-ASK1) is shown in red and by a star (*). Three autophosphorylation sites identified in ASK1 are enclosed in squares.

ASK1 was first identified as a member of the MAPKKK family and was found to activate the MAPKK 4 (MKK4)-JNK and MKK3/6-p38 pathways but not the MAP/ERK kinase (MEK)-extracellular signal-regulated kinase (ERK) pathway (Figure 1). *In vitro *kinase assays revealed that ASK1 activated MKK3/p38- and MKK4/JNK-pathways, and directly phosphorylated MKK3, MKK4 and MKK6. Furthermore, overexpression of ASK1 wild-type or a constitutively active mutant induced apoptosis in a fetal lung cell line [2,3]. Invertebrate orthologs of mammalian ASK family members have been identified in *Drosophila melanogaster *and *Caenorhabditis elegans*, designated as DASK1 and NSY-1, respectively, indicating that ASK-MAPK cascades are evolutionarily well conserved among species (Figure 2) [4-6].

#### Homo-oligomerization and signalosome

Previous studies have revealed that ASK1 is activated by various stresses, including oxidative stress, endoplasmic reticulum (ER) stress and calcium influx [3,7,8]. Phosphorylation of Thr845 in mouse ASK1 is essential for the activation of ASK1 [9]. Thr845 is located in the activation loop of the kinase domain and is one of the autophosphorylation sites (Figure 2) [10]. Under nonstress conditions, ASK1 forms a high molecular mass complex, designated as the ASK1 signalosome [11]. In the signalosome, ASK1 is homo-oligomerized through the C-terminal coiled-coil domain (CCC). Following hydrogen peroxide stimulation, Thr845 appears to be autophosphorylated *in trans*, thereby leading to ASK1 activation. Although an ASK1 mutant lacking the CCC (ASK1ΔCCC) exhibited lower basal kinase activity compared to wild-type, artificially homo-oligomerized ASK1ΔCCC was autophosphorylated and activated without any stimulation. These results suggest that ASK1 homo-oligomerization is crucial to the activation of ASK1 [9].

#### Reciprocal regulation by thioredoxin and TRAFs

The first identified ASK1-interacting molecule was thioredoxin (Trx), which turned out to play an important role in oxidative stress-induced regulation of ASK1. Trx is an oxidoreductase, which has a dithiol-disulfide active site and is thought to have anti-apoptotic effects [12]. A reduced but not oxidized form of Trx interacts noncovalently with the N-terminus of ASK1 and suppresses ASK1 kinase activity at the basal level by inhibiting N-terminal proximity between ASK1 molecules. ASK1ΔN, which is an N-terminal truncated mutant of ASK1 lacking a Trx binding region, is thus a constitutively active mutant and more potently induces apoptosis in various cultured cells [3,13,14]. Interaction between Trx and ASK1 is observed only under reducing conditions, and Trx is oxidized and dissociates from ASK1 when cells are exposed to oxidative stress such as reactive oxygen species (ROS) [3]. The ROS-dependent dissociation of Trx from ASK1 thus serves as a molecular switch, which converts oxidative stress to a phosphorylation-dependent signal.

Tumor necrosis factor-α receptor-associated factors (TRAFs) are also important in the regulation of ASK1 activity. The interactions between TRAF family proteins and ASK1 were first examined because tumor necrosis factor-α (TNF-α induced ASK1 activation [2]. Among the TRAF family proteins, TRAF1, TRAF2, TRAF3, TRAF5 and TRAF6 associate with ASK1, but only TRAF2, TRAF5 and TRAF6 increase ASK1 kinase activity [15]. TRAF2 and TRAF6 have been shown to be recruited to ASK1 in an ROS-dependent manner [11,16]. In mouse embryonic fibroblasts (MEFs) derived from TRAF2- or TRAF6-deficient mice, the activation of ASK1 induced by hydrogen peroxide was dramatically attenuated and both JNK and p38 activities were also reduced [11]. These results suggest that TRAF2 and TRAF6 play essential roles in the ROS-dependent activation of ASK1.

As described above, ASK1 forms a large molecular mass complex, the ASK1 signalosome, which is required for ASK1 activity. In response to hydrogen peroxide or TNF-α, the ASK1 signalosome forms an even higher molecular mass complex. In TRAF6-deficient MEFs, ASK1 signalosome no longer exhibited the molecular shift induced by hydrogen peroxide, suggesting that TRAF6 (and presumably TRAF2) is required for ROS-induced formation of the activated ASK1 signalosome [11].

Taken together, these findings indicate that in nonstress conditions ASK1 is oligomerized through its CCC but it remains in an inactive form by the suppressive effect of Trx [9]. Upon ROS stimulation, Trx dissociates from ASK1, while TRAF2 and TRAF6 are recruited to ASK1 to form a larger molecular mass complex. Subsequently, ASK1 forms homo-oligomeric interactions through not only the CCC but also the N-terminal coiled-coil domain (NCC), which leads to the fully activated form of ASK1 [13,16].

#### Negative regulation by phosphatases

On the other hand, two protein phosphatases have been reported to inactivate ASK1 by dephosphorylation of Thr845 in ASK1. Protein phosphatase 5 (PP5) recognizes activated ASK1 in response to ROS and dephosphorylates Thr845. This mechanism functions as a negative feedback after the activation of ASK1 [17]. Another serine/threonine protein phosphatase, PP2Cε, has also been found to be a negative regulator of ASK1 under basal conditions. Endogenous PP2Cε interacts with ASK1, and *in vitro *experiments revealed that PP2Cε retains ASK1 in the inactive form by directly dephosphorylating Thr845. In response to hydrogen peroxide or TNF-α treatment, PP2Cε dissociates transiently from ASK1, which may contribute to ASK1 activation upon these stimuli [18].

#### Negative regulation by 14-3-3

The 14-3-3 family consists of highly conserved multifunctional phospho-serine/phospho-threonine binding proteins that participate in the regulation of diverse intracellular signal transduction systems, including survival and apoptotic signaling [19]. In ASK1 regulation, 14-3-3 proteins bind to ASK1 via a site involving Ser966 of ASK1 in a phospho-dependent manner and contribute to the suppression of ASK1 kinase activity under nonstress conditions. ASK1 S966A, which could not bind to 14-3-3 proteins, exhibited higher kinase activity than the wild-type, and the overexpression of ASK1 S966A enhanced cell death relative to the overexpression of the wild-type without any stimulation in cultured cells [20]. Dephosphorylation of ASK1 at Ser966 was induced by hydrogen peroxide treatment and the dissociation of 14-3-3 from ASK1 correlated with the dephosphorylation, resulting in the enhancement of ASK1 catalytic activity [21]. Recently, PP2A has been suggested to be a phosphatase responsible for the TNF-α-dependent dephosphorylation of ASK1 at Ser966 in vascular endothelial cells. PP2A forms a complex with apoptosis signal-regulating kinase1-interacting protein 1 (AIP1) under nonstress conditions. Upon TNF-α treatment, AIP phosphorylation is mediated by the Serine/Threonine protein kinase receptor-interacting protein (RIP1), and AIP is recruited to ASK1 together with PP2A. Therefore, ASK1 phospho-Ser966 is dephosphorylated by PP2A, which leads to the dissociation of 14-3-3 proteins from ASK1, resulting in the activation of the ASK1-JNK pathway [22-24].

### The roles of ASK1 in stress responses

#### TNF signaling

TNF-α treatment induces JNK activation in a TRAF2-dependent manner [15,25]. The requirement of ASK1 for TNF-α-induced JNK activation has been demonstrated. The expression of a dominant-negative mutant of ASK1 (ASK1 K709R) inhibited the JNK activation induced by TNF-α stimulation or TRAF2 overexpression in a dose-dependent manner [15]. Furthermore, overexpression of an ASK1 dominant-negative mutant reduced the DNA fragmentation induced by TNF-α, suggesting that ASK1 is necessary for TNF-α-dependent apoptosis [2]. ASK1 interacts with TRAF2 not only by TNF-α treatment but also by hydrogen peroxide treatment [11,15]. Experiments with mouse embryonic fibroblasts (MEFs) derived from ASK1-deficient mice revealed that ASK1 was necessary for the sustained activation of JNK and p38 induced by TNF-α or hydrogen peroxide. Moreover, in ASK1-deficient MEFs, apoptosis induced by TNF-α or hydrogen peroxide was suppressed. Treatment with the antioxidant N-acetylcysteine (NAC) suppressed the TNF-α-induced ASK1 activation, the sustained activation of JNK and apoptosis, suggesting that the generation of ROS is required for the TNF-α-induced ASK1-JNK axis and apoptosis [3,26].

#### Fas signaling

**Figure 3:**
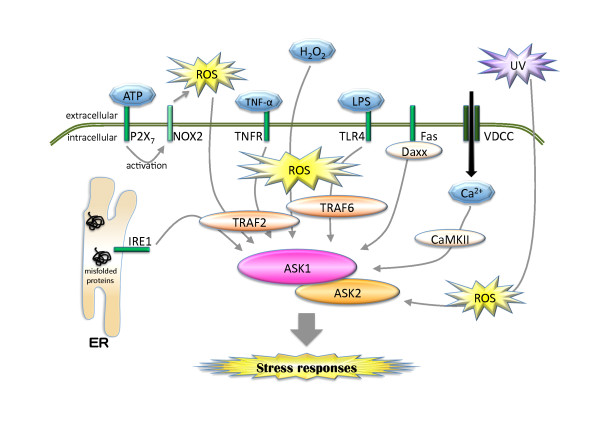
**Overview of signaling upstream of ASK1 and ASK2**. Various stimuli activate ASK1 and ASK2. In some of these stimuli, ROS generation is required to activate the ASK family. The recruitment of TRAF2 and/or TRAF6 to ASK1 is also important in the activation. ASK1 and ASK2 can form a complex in the cell. For details, see text.

On the other hand, an agonistic antibody to Fas that activates the Fas death receptor also induces ASK1 activation through the association between ASK1 and Daxx. ASK1 has been thought to be necessary for the subsequent JNK and p38 activation, because their activation levels after Fas stimulation were reduced in ASK1-deficient thymocytes (Figure 3) [26,27]. However, apoptosis induced by Fas antibody was unaffected in the ASK1-deficient thymocytes, suggesting that ASK1 is not required for Fas-induced apoptosis in these cells [26].

#### TLR4 signaling

Recently, ASK1's critical role in innate immunity has been elucidated. Toll-like receptor-4 (TLR4) recognizes lipopolysaccharide (LPS), one of the major pathogen-associated molecular patterns of Gram-negative bacteria, as a ligand and initiates signal transduction pathways that are important for innate immunity (Figure 3) [28]. In dendritic cells and splenocytes derived from ASK1-deficient mice, LPS-induced activation of p38 and production of inflammatory cytokines such as interleukin-6 (IL-6) and TNF-α were significantly inhibited, suggesting that ASK1 is required for TLR4 signal transduction. In a manner similar to the case with TNF-α, pre-treatment with antioxidants such as NAC and propyl gallate reduced LPS-induced activation of both ASK1 and p38, and also reduced the production of IL-6 in RAW264.7 macrophage-like cells [15,26,29]. Intriguingly, ASK1-deficient mice were resistant to LPS-induced septic shock. The phenotype can be explained by the fact that the serum concentrations of TNF-α and nitric oxide (NO), principal factors responsible for septic shock, were reduced in ASK1-deficient mice, presumably by the attenuation of TLR4 signaling [29].

#### Calcium signaling

Calcium signaling also activates the ASK1-p38 pathway. In neuronal cells, the intracellular calcium ion concentration and the activity of calcium/calmodulin-dependent protein kinase (CaMK) play pivotal roles in the regulation of neuronal development, plasticity and behavior [30]. In primary neurons derived from ASK1-deficient mice, p38 activation induced by calcium influx, which was evoked by treatment with KCl or maitotoxin, was significantly impaired [31]. FPL64176, a voltage-dependent calcium channel (VDCC) agonist, activated ASK1 and p38, which was attenuated by CaMKII inhibitor KN93, suggesting that calcium signaling activates the ASK1-p38 pathway through CaMKII activation. CaMKII was able to activate ASK1 by increasing the phosphorylation of Thr845 in cultured cells, although CaMKII failed to directly phosphorylate Thr845 of ASK1 *in vitro*. The precise mechanism of how CaMKII activates ASK1 remains to be elucidated (Figure 3) [8].

#### Extracellular ATP signaling

Recently, the involvement of the ASK1-p38 pathway in ATP-dependent macrophage apoptosis has been elucidated [32]. Besides its role as a primary source of energy in the body, extracellular ATP acts as an intercellular signal transmitter. It has already been well established that high concentrations of ATP (~mM) induce apoptosis in hematopoietic cells [33,34]. ATP receptors include two subtypes: P2X and P2Y. P2X are channel complexes, whereas P2Y are G-protein coupled heptahelical receptors [35,36]. Treatment with ATP induced p38- and ROS-dependent apoptosis in RAW264.7 cells, which was suppressed by the P2X_7 _inhibitor KN62. In ASK1-deficient macrophages, ATP-induced p38 activation and subsequent apoptosis were reduced, whereas ROS production was not reduced, suggesting that the activation of the ASK1-p38 pathway was crucial to the mediation of ATP-dependent apoptosis (Figure 3). It is well known that nicotinamide adenine dinucleotide phosphate (NADPH) oxidase and mitochondria are two major ROS generators in cells. In this case, the NOX2 knockdown, which is highly expressed in macrophages among the members of the NADPH oxidase family, resulted in the reduction of ROS production and the attenuation of subsequent p38 activation and apoptosis induced by ATP. NADPH oxidases produce ROS toward the extracellular environment. Thereafter, ROS is considered to enter the cytosol, which leads to ASK1 activation. Taken together, these findings suggest that extracellular ATP binds to P2X_7_, and that subsequent ROS-dependent activation of the ASK1-p38 pathway appears to induce apoptosis in macrophages (Figure 3) [32].

### The function of ASK2 in tumorigenesis

Shortly after the identification of ASK1, ASK2 (also designated as MAP3K6) was identified as an ASK1 binding protein using a yeast two-hybrid screen [37]. ASK2 is highly related to ASK1, especially in the kinase domain, and associates with ASK1 endogenously (Figure 2) [38]. While ASK1 is ubiquitously expressed in various tissues, ASK2 is specifically expressed in tissues that are directly exposed to the external environment of the body, such as skin, lung and the gastrointestinal tract [39]. Intriguingly, ASK2 protein could not be detected in MEFs or in bone marrow-derived macrophages (BMDM) derived from ASK1-deficient mice, even though the ASK2 mRNA expression level was slightly higher than in wild-type cells. Treatment with a proteasome inhibitor rescued endogenous ASK2 protein from degradation in ASK1-deficient MEFs. A similar restorative effect was observed by the exogenous expression of ASK1 wild-type or even by that of kinase-negative mutant in ASK1-deficient MEFs. These data suggest that ASK1 prevents ASK2 from degradation by interacting with it in a kinase activity-independent mechanism. Although exogenously overexpressed ASK2 possesses very low kinase activity, co-expression with the ASK1 kinase-negative mutant confers a strong kinase activity and the ability to induce JNK and p38 activation to ASK2. Conversely, ASK1 phosphorylation at Thr845 can be achieved by ASK2 kinase activity in the ASK1-ASK2 heteromeric complex, eventually leading to ASK1 activation [38].

Recently, a novel function of ASK2 in skin tumorigenesis has been elucidated along with the complex relationship between ASK2 and ASK1 [39]. It has been suggested that ROS accumulation is relevant to tumor initiation and promotion [40]. Also, an increasing number of studies discuss the relationship between tumorigenesis and inflammation. ASK1 is involved in the induction of apoptosis, which is assumed to be a crucial tumor suppression mechanism. ASK1 is also required for the ROS-dependent induction of proinflammatory cytokines [29].

One of the most analyzed inflammation-associated tumor models is the two-step "initiation and promotion" scheme for the induction of mouse skin tumor [41]. In this model, tumor initiation is evoked by a low dose of a genotoxic carcinogen such as 7.12-dimethylbenz(a)anthracene (DMBA), which does not develop cancer by itself. Tumor formation can be promoted only when a tumor-promoter such as 12-O-Tetradecanoylphorbol-13-acetate (TPA) is repeatedly applied to the mouse afterwards. In this model system, the number of papillomas was significantly higher in ASK2-deficient mice than in wild-type mice [39].

In ASK2-deficient keratinocytes, the activation of p38 and that of JNK induced by DMBA through ROS production were reduced, and subsequent caspase-3 activation and apoptosis were also attenuated. Similarly, UV-A irradiation-triggered activation of p38 and JNK, which is mediated by ROS accumulation, was suppressed in ASK2-deficient keratinocytes. Subsequent cell death was also attenuated. These results suggest that ASK2 appears to serve as a tumor suppressor through the induction of MAPK activation and subsequent apoptosis in ROS-accumulated damaged cells. Immunohistochemical staining of human epithelial cancer tissue provided the evidence that ASK2 functioned as a tumor suppressor protein. That is, the ASK2 protein expression level was significantly reduced in the esophageal squamous cell carcinoma (ESCC) region in epithelial tissue, although ASK2 expression was throughout the normal cell regions [39].

**Figure 4:**
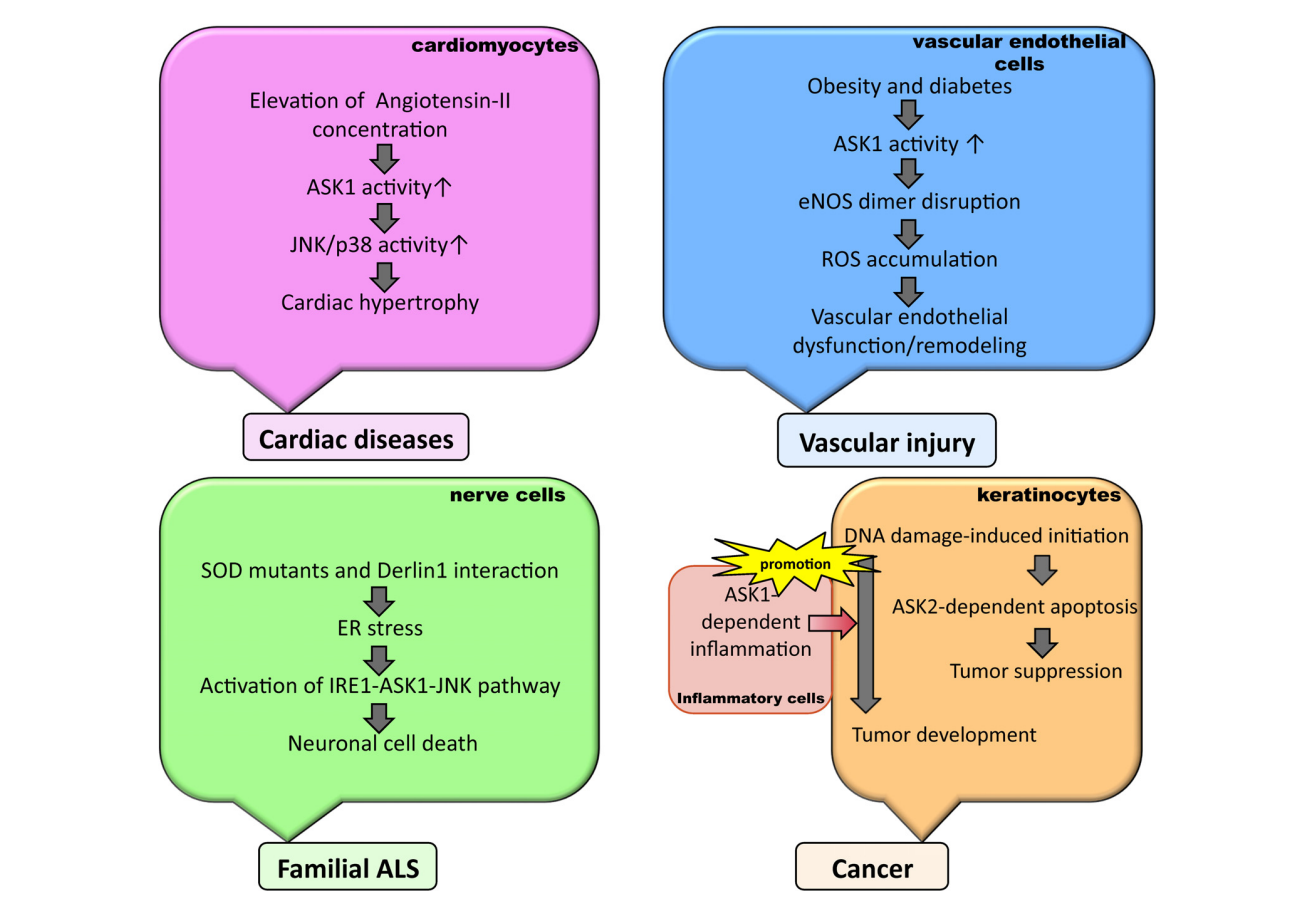
**Proposed mechanisms of pathogenesis of ASK family-related diseases**. ASK family proteins are involved in the pathogenesis of various diseases. Detailed roles of the ASK family in the development of individual diseases are described in the text.

On the other hand, in the skin tumorigenesis model, fewer papillomas were induced in ASK1 and ASK2 double-deficient mice than in ASK2-deficient mice, indicating that ASK1 counteracts to ASK2 in tumorigenesis. It has become clear that ASK1 is required for the production of inflammatory cytokines such as TNF-α and IL-6 in skin induced by treatment with TPA. In summary, ASK1 and ASK2 play opposite roles in this tumorigenesis model. That is, ASK2 functions as a tumor suppressor by eliminating damaged cells through apoptosis in the initiation stage; conversely, ASK1 functions as a tumor promoter in the promotion stage by inducing inflammation (Figure 4) [39].

### ASK1 in cardiovascular diseases

In various cardiovascular diseases, including hypertension, myocardial infarction and diabetic cardiomyopathy, individual cardiomyocytes increase in size to compensate in cardiac functions and damaged heart tissue, in a phenomenon called cardiac hypertrophy. Although cardiac hypertrophy is an adaptive response to the initial changes in cardiac diseases, sustained hypertrophy potentially causes cardiac morbidity and mortality. Ventricular hypertrophy is characterized by myocyte gene reprogramming and by the accumulation of extracellular matrix (ECM) proteins, which play crucial roles in ventricular fibrosis and remodeling [42,43].

Angiotensin II (AngII), the central product of the renin-angiotensin system, is known to play a key role in cardiovascular diseases [42]. In cardiac myocytes, ASK1 is activated by AngII via angiotensin II type I receptor (AT1) in an ROS-dependent manner and is involved in the induction of cardiac hypertrophy, which is mediated at least in part by the activation of JNK and p38 (Figure 4) [44,45]. In ASK1-deficient mice, AngII-induced activation of JNK and p38 was inhibited compared to that in wild-type mice. Moreover, cardiac hypertrophy induced by AngII was significantly suppressed in ASK1-deficient mice [44]. These data suggest that ASK1 plays an important role in AngII signaling, leading to cardiac hypertrophy.

In obese and diabetic mice, olmesartan, an AT1 blocker, notably prevented cardiac inflammation, cardiac interstitial fibrosis, coronary arterial remodeling and vascular endothelial dysfunction, suggesting that AngII signaling is also involved in vascular diseases [46]. High-fat-diet-induced diabetes provokes vascular endothelial dysfunction following the disruption of the endothelial nitric oxide synthase (eNOS) dimer in arteries. Although the precise mechanism is unclear, the disruption of the eNOS dimer contributes to the generation of superoxide [47-50]. In ASK1-deficient mice, the impairment of vascular endothelial function by diet-induced diabetes was suppressed. This beneficial phenotype was associated with the attenuation of eNOS dimer disruption and the subsequent reduction in superoxide production. It is conceivable that ASK1 may be activated in diabetic mice by an Ang II-dependent mechanism and that it accelerates eNOS disruption, resulting in superoxide accumulation and thus the induction of vascular endothelial dysfunction and remodeling (Figure 4) [46].

Moreover, the inhibition of ASK1 by the recombinant adeno-associated virus (rAAV) expressing a dominant-negative ASK1ΔN-KR mutant was capable of suppressing heart failure progression by preventing cardiomyocyte apoptosis even after the onset of hereditary cardiomyopathy [51]. These results suggest that ASK1 is a promising drug target for the treatment of a variety of cardiovascular diseases.

### ASK1 in neurodegenerative diseases

Recently, endoplasmic reticulum (ER) stress, which also activates ASK1, has been suggested to be involved in a variety of neurodegenerative diseases [52]. The ER is the organelle where secretory or transmembrane proteins are synthesized and folded into an appropriate configuration. The ER also has a quality-control function with these proteins; only correctly folded proteins are excreted from the ER, while unfolded or misfolded proteins are degraded via endoplasmic reticulum-associated protein degradation (ERAD), which is mediated by the ubiquitin proteasome system (UPS). The accumulation of unfolded or misfolded proteins in the ER is one of the major causes of ER dysfunction [53]. These anomalous proteins induce oligomerization-dependent auto-phosphorylation of PERK and IRE1, which are transmembrane serine-threonine kinases localized to the ER membrane, and initiate ER stress signaling from the ER to the cytosol [54,55]. Therefore, to prevent ER stress, it is important to preserve normal ERAD function. The malfunction of the ERAD itself is thought to contribute to the development of ER stress and subsequent aggravation of several neurodegenerative diseases [52].

Several kinds of inherited neurodegenerative diseases are caused by an expanded polyglutamine (polyQ) chain in specific proteins. For example, polyQ-expanded Huntingtin and Ataxin-1 are observed in Huntington's disease and spinocerebellar ataxia type-1 (SCA-1), respectively. These abnormally expanded polyQ-containing proteins cannot fold correctly, resulting in the formation of insoluble intracellular inclusions [56]. The pathogenic length polyQ (Q79) derived from carboxy-terminal fragments of the SCA-3 protein, but not normal length polyQ (Q14), induced ER stress by impairing proteasome activity [57]. Under such ER stress conditions, the IRE1-TRAF2-ASK1 complex was formed on the ER membrane, leading to the activation of the ASK1-JNK pathway. In ASK1-deficient primary neurons, JNK activation and cell death induced by the expression of expanded polyQ are reduced, suggesting that ASK1 is involved in ER stress-dependent cell death through the activation of JNK [7].

Recently, it has been revealed that ASK1 is also involved in motor neuron cell death in familial amyotrophic lateral sclerosis (ALS). To date, Cu/Zn superoxide dismutase 1 (SOD1) has been thought to be involved in the majority of familial ALS, because multiple hereditary SOD point mutations cause motor neuron death through various cellular events in diverse ways. However, the precise mechanism underlying these events is still unknown [58]. In a recent study, Derlin-1, an important component of the ERAD machinery, which constitutes the retro-translocon of the ER, interacted with SOD mutants but not with wild-type SOD1. Overexpression of SOD mutants inhibited ERAD and thereby triggered ER stress with the activation of the IRE1-TRAF2-ASK1 pathway [59]. In the *SOD1*^*G*93*A *^gene transgenic mouse, which exhibits a disease phenotype similar to that of familial ALS patients, the accumulation of SOD1 G93A and the interaction between Derlin-1 and SOD1 G93A were observed along with the onset of the disease [60]. The region where Derlin-1 interacts with the SOD1 mutants was narrowed down to only 12 amino acids (designated as CT4). CT4 overexpression led to the dissociation of SOD1 mutants from Derlin-1, presumably through the competitive interaction with SOD1 mutants, and consequently IRE1, PERK and ASK1 activation was attenuated in the cells. Compared with *SOD1*^*G*93*A *^transgenic mice, *SOD1*^*G*93*A *^transgenic mice crossed with ASK1 -/- mice, in which the neuronal cell death in spinal cords was ameliorated, survived longer [59].

In summary, SOD1 mutants trigger ER stress by anomalous interaction with Derlin-1, and the subsequent activation of ASK1 is involved in the progression of familial ALS. Therefore, inhibitors of the upstream signaling pathway of ASK1, such as Derlin-CT4, or inhibitors of ASK1 itself, might be therapeutic for ALS patients [59].

In addition, it has been suggested that ASK1 may contribute to the pathology of Alzheimer's diseases and Parkinson's disease through the induction of neuronal cell death [61,62]. Thus, ASK1 has the potential to become a therapeutic target for these neurodegenerative diseases.

## Conclusion

ASK1 is a member of the MAPKKK family in a dynamic protein kinase network. ASK1 plays significant roles in diverse stress responses. ASK1 also plays key roles in multiple diseases, including cancer, cardiovascular diseases and neurodegenerative diseases. ASK2 is also a unique protein kinase that functions by forming a heteromeric complex with ASK1 but plays an opposite role to ASK1 in tumorigenesis. It is important to identify the similarities and differences among ASK family proteins, including their activation mechanisms, in order to understand the specific roles of the individual proteins. Considering the involvement of ASK family proteins in critical diseases at the present time, further studies on the detailed regulatory mechanisms of the ASK family are crucial for the development of novel therapeutic strategies.

## Competing interests

The authors declare that they have no competing interests.

## Authors' contributions

KH wrote the manuscript and designed the figures. IN and CR contributed to improve the contents of the document. HI supervised the process of writing.
